# CD28: Direct and Critical Receptor for Superantigen Toxins

**DOI:** 10.3390/toxins5091531

**Published:** 2013-09-09

**Authors:** Raymond Kaempfer, Gila Arad, Revital Levy, Dalia Hillman, Iris Nasie, Ziv Rotfogel

**Affiliations:** Department of Biochemistry and Molecular Biology, Institute of Medical Research Israel-Canada, Hebrew University-Hadassah Medical School, Jerusalem 91120, Israel; E-Mails: sh_arad@zahav.net.il (G.A.); revital.levy@mail.huji.ac.il (R.L.); daliabh@gmail.com (D.H.); iris.nasie@gmail.com (I.N.); rotfogel@gmail.com (Z.R.)

**Keywords:** superantigen toxins, inflammatory cytokine storm, lethal toxic shock, biodefense, CD28 receptor, CD28 dimer interface

## Abstract

Every adaptive immune response requires costimulation through the B7/CD28 axis, with CD28 on T-cells functioning as principal costimulatory receptor. Staphylococcal and streptococcal superantigen toxins hyperstimulate the T-cell-mediated immune response by orders of magnitude, inducing a lethal cytokine storm. We show that to elicit an inflammatory cytokine storm and lethality, superantigens must bind directly to CD28. Blocking access of the superantigen to its CD28 receptor with peptides mimicking the contact domains in either toxin or CD28 suffices to protect mice effectively from lethal shock. Our finding that CD28 is a direct receptor of superantigen toxins broadens the scope of microbial pathogen recognition mechanisms.

## 1. Introduction

Superantigens are pyrogenic exotoxins secreted by the ubiquitous Gram-positive bacterial strains, *Staphylococcus aureus* and *Streptococcus pyogenes*. The superantigens form a broad family of well over three dozen structurally diverse toxins that are highly lethal to humans. Thus, staphylococcal enterotoxin B (SEB), a prominent staphylococcal superantigen, is only 27% homologous to the related enterotoxin SEA, and shares only 6% sequence homology with staphylococcal toxic shock syndrome toxin-1 (TSST-1), the most remote member of the staphylococcal superantigen family [1]. This sequence diversity results in structural differences that render broad vaccine development virtually impossible.

Superantigens are among the most stable proteins known: they resist heat and survive boiling, protease digestion, as well as acid denaturation. Indeed, even at very low concentrations, SEB can cause severe food poisoning and incapacitation [2]. Buildup of higher concentrations of toxin, as occurs during staphylococcal or streptococcal infections, will lead to toxic shock, with lethal consequences [3]. The extraordinary combination of stability, ease of production as natural mixtures from bacterial cultures, and high toxicity to humans renders the superantigen toxins a potential biological weapon [3]. Indeed, the United States government weaponized SEB (now known to be a mixture of related superantigen toxins) prior to 1969, when this practice was stopped [2]. Nonetheless, the superantigen toxins are classified as a Category B priority pathogen and remain a major biological terror threat today, defining a need for broadly effective antidotes that target the toxins and block their action. That said, the main actual threat of superantigen toxins is in the clinic, as causative agents of toxic shock and often implicated in hospital deaths resulting from nosocomial infections.

Superantigen toxins can evoke a T-cell-mediated immune response that is up to four orders of magnitude greater than that induced by ordinary antigens, leading to a massive inflammatory reaction that is lethal [4]. Pioneering work by Marrack and others in the late 1980s and 1990s showed that the superantigen achieves this by binding directly, as intact protein, to the major histocompatibility class II (MHC-II) molecule on the antigen-presenting cell and the T-cell receptor (TCR) on the T-cell, without need for antigen processing and bypassing the restriction typical for conventional antigens [5,6,7]. In this manner, the superantigen provides an intermolecular bridge between the MHC-II molecule and the TCR. This classical view held for over two decades, until the recent discovery that in order to induce a potent, harmful inflammatory response, the superantigen toxin must bind, in addition, directly to a third receptor. This critical receptor is CD28 [8], hitherto unknown to bind pathogens or virulence factors and thought to function only as the immune system’s principal costimulatory receptor.

Every immune response requires costimulation through the B7/CD28 costimulatory axis. As the principal costimulatory receptor, CD28 is a critical regulator of the immune response [9,10,11]. Expressed constitutively on T cells, CD28 is a homodimer that interacts with its B7 coligands to transduce the signal essential for an immediate T cell response [10,11,12,13]. The CD28 coligand B7-2 (CD86) is expressed constitutively whereas B7-1 (CD80) is expressed only upon antigenic stimulation [13,14]. Therefore, signaling by antigens early in the immune response is controlled by the interaction of CD28 with B7-2 [15,16]. Apart from its B7 coreceptors, CD28 was not known to engage other ligands.

As will be reviewed below, a number of studies suggested that like ordinary antigens, the superantigens also require costimulation. Interpreted in support of conventional costimulation, these observations taught away from the very idea that CD28 could be a direct receptor for superantigen toxins, let alone a receptor critical for the action of the toxin. The path to the discovery that CD28 is an indispensable superantigen toxin receptor to which superantigens must bind directly was long and arduous [1,8]. Here, we recount how that insight was achieved after decades in which only the MHC-II and TCR molecules were believed to transmit superantigen signaling. We now know that blocking access of the superantigen to CD28 is enough to provide protection from lethal toxic shock [8].

## 2. Results and Discussion

### 2.1. The Classical View of Superantigen Receptor Engagement

**Figure 1:**
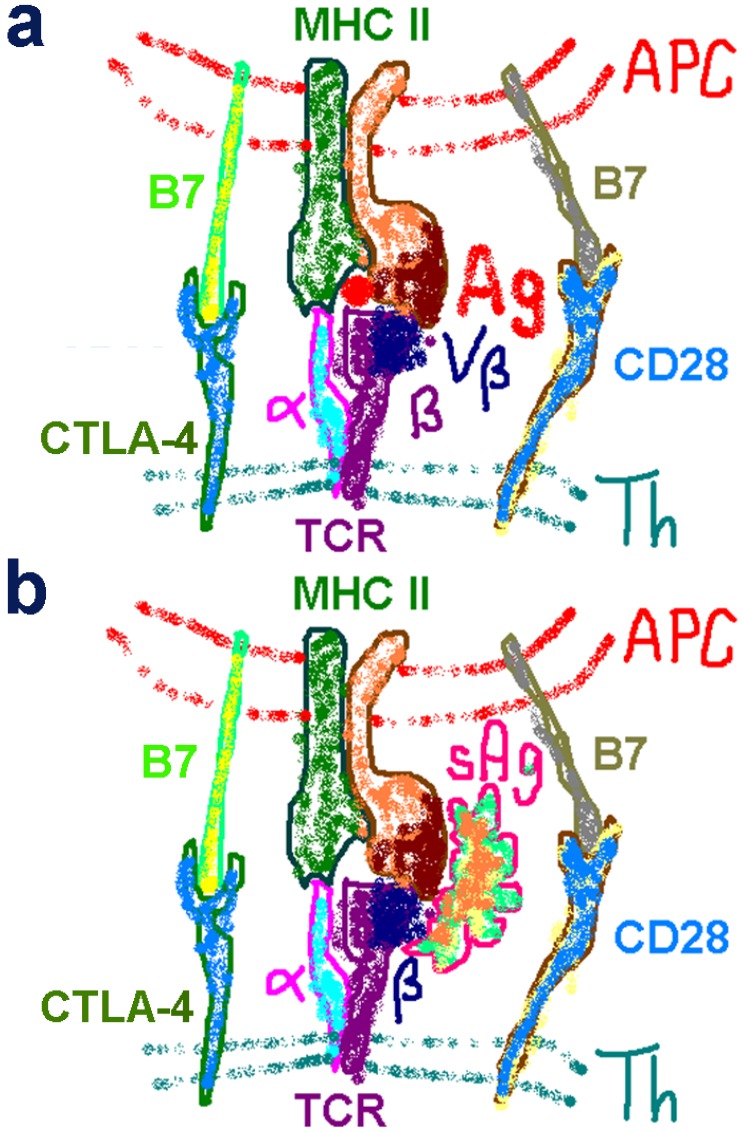
Classical views of antigen and superantigen receptor engagement. (**a**) Schematic of receptor engagement by conventional antigens. The processed antigen (Ag, red dot) is presented by the MHC class II molecule expressed on the antigen-presenting cell (APC) to the T-cell receptor (TCR) expressed on the helper T (Th) cell. Only α- and β-chains of the TCR are shown for simplicity; Vβ denotes the variable region of the β-chain. Positive control is provided through B7/CD28 costimulatory receptor signaling and negative control through B7/CTLA-4 costimulatory receptors; CD28 and CTLA-4 function as homodimers. (**b**) Schematic of classical receptor engagement by superantigens. The intact superantigen (sAg) is presented without processing outside the cell to MHC class II molecule and TCR, engaging its Vβ chain. CD28 and CTLA-4 serve their conventional costimulatory function.

The cartoon of Figure 1a summarizes the major elements involved in an immune response induced by conventional antigens. The antigen-presenting cell (APC) presents the processed antigen through a pocket in its MHC-II molecule to a specific Vβ chain on the TCR expressed on the surface of very few T helper (Th) cells. Costimulation is provided by the homodimeric CD28 receptor which engages its B7 coligands. Later, CTLA-4 is expressed and acts as a negative costimulatory receptor by competing more effectively than CD28 for the B7 coligands, thus terminating the immune response.

The early, 1990s view of superantigen action is illustrated in Figure 1b. Unlike a conventional antigen, the superantigen engages both MHC-II and TCR from the outside as an intact, unprocessed protein. Binding to a variety of MHC-II and Vβ chains, it activates a high percentage of Th cells. All superantigens function in this manner, but individual superantigens interact distinctly with the two receptors. Costimulation is thought to be provided by B7/CD28 signaling, as for ordinary antigens.

The understanding that like conventional antigens, superantigens require B7/CD28 costimulation was based on a number of observations. CD28 knockout mice proved resistant to lethal superantigen challenge [17,18]. Monoclonal [8] or polyclonal [19] antibodies against B7-2, but not against B7-1, inhibited the induction of *IL2* and *IFN-γ* genes in human PBMC [8] and systemic IL2 release [19] and protected mice from the lethal effect of SEB [19]. Staphylococcal enterotoxin-induced expression of the *IL2* gene is regulated coordinately through signals transduced through the TCR and through CD28, a finding that was interpreted in support of an essential need for costimulation [20]. Among other coreceptors, CD28 costimulatory signaling was implicated in SEB action through the inhibitory effect of anti-CD28 antibodies on the induction of TNF-α and IFN-γ [21]. Together, these results provided a coherent body of evidence, strongly suggesting that the role of CD28 in superantigen action was merely to provide costimulation as in the case of conventional antigens. They taught away from the concept that CD28 could function distinctly, as a direct and crucial superantigen receptor.

### 2.2. A Novel and Critical Domain in Superantigens of Unknown Function

The powerful ability of superantigens to activate T cells involves their tight binding to the TCR and the MHC class II molecule, stabilized by interactions at multiple sites [7,22,23,24,25]. In an effort to develop an antagonist to superantigen toxins, we synthesized short peptide mimetics of regions in SEB that were known to contact either the MHC-II molecule, the TCR, or both [1]. In addition, we synthesized peptides containing SEB residues 150–161, which forms a β-strand/hinge/α-helix domain that is conserved among superantigens yet is not known to be involved in the binding of TCR or MHC-II molecules [7,22,23,24,25]. None of the peptides chosen to target the binding sites in superantigens for MHC-II and/or TCR reduced the induction of human *IL2* or *IFN-γ* genes in human PBMC whereas by contrast, peptides mimicking the SEB 150–161 domain were strong inhibitors of the staphylococcal toxins SEB, SEA, and TSST-1, as well as of streptococcal pyrogenic exotoxin A (SPEA) [1]. These broadly effective peptides, including some sequence variants we created, were active *in vivo*, protecting mice from lethal challenge with each of these toxins and even were capable of rescuing mice several hours after they had been exposed to a superantigen toxin [1,26]. Remarkably, a single dose of peptide given at the first superantigen challenge not only protected the mice but rendered them fully resistant to further toxin challenges with the same toxin or even different ones [1,26], and adoptive transfer of their serum rendered naïve mice toxin-resistant [1] owing to the presence of broadly protective antibodies [27]. Thus, when the peptide blocked the lethal action of the toxin, it allowed the superantigen vigorously to induce humoral immunity against itself [1,26,27].

**Figure 2:**
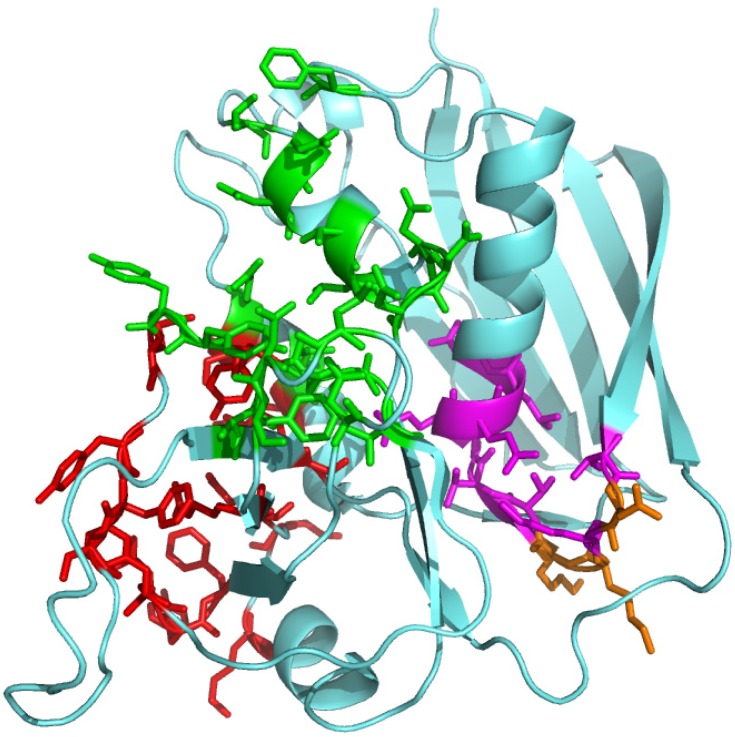
Structure of the superantigen molecule and its CD28 binding domain. In the SEB structure (pdb3seb.ent), amino acid residues contacting MHC-II are colored red; those contacting the TCR are colored green. Residues 150–161 in SEB (TNKKKVTAQELD) form the conserved β-strand(8)/hinge/α-helix(4) domain [1] that is shown in magenta, with solvent-accessible residues [28] N151, K153 and K154 in orange. The β-strand(8)/hinge/α-helix(4) domain is accessible to a third receptor, CD28 [8].

These biological data attracted strong attention in the US biodefense community [2]. They defined a novel superantigen domain that is critical for T-cell activation, yet left the function of this domain completely unresolved. Inspection of Figure 2 will show that the conserved β-strand/hinge/α-helix domain (denoted by magenta and orange residues) resides in SEB opposite the side that engages the MHC-II molecule and the TCR (denoted by red and green residues, respectively) and thus is fully accessible to another ligand. We speculated, therefore, that the β-strand/hinge/α-helix domain might engage an as yet unknown, third receptor essential for superantigen activity. But what could that receptor be? It would take a long and indirect experimental path to show that it is CD28.

### 2.3. The Novel Superantigen Domain Engages CD28

As said, various peptide mimetics of the 12-amino acid β-strand/hinge/α-helix domain in SEB strongly attenuated the induction of Th1-type inflammatory cytokine genes encoding IL2, IFN-γ and TNF [1,8]. The first hint in our search for the novel superantigen receptor was provided by the observation that such a peptide will also inhibit the induction of these genes in human PBMC in the absence of a superantigen, by anti-CD28/anti-CD3 monoclonal antibody treatment, a model for the normal T cell-mediated immune response, yet not the induction by anti-CD3 alone [8]. This showed that the peptide was not blocking signaling through the TCR. Indeed, induction of *IL2* and *IFN-γ* genes by a monoclonal antibody against CD28 alone was also sensitive to inhibition by the peptide [8]. Yet, the peptide did not bind to this antibody [8]. This raised the intriguing possibility that the superantigen mimetic peptide somehow competed with the specific anti-CD28 monoclonal antibody for its binding site in CD28. If that were so, then possibly the intact superantigen molecule might have a binding site in CD28.

To evaluate this possibility, we first screened a random 12-mer phage display peptide library for peptides having the ability to bind to immobilized recombinant CD28 and then to be displaced by SEB. We argued that if SEB were to bind to CD28, a random peptide capable of binding to the same site in CD28 might be displaced by the superantigen from CD28. We isolated a few of such displaced peptides; they were highly enriched for SEB antagonists that strongly inhibited SEB-mediated induction of *IL2* and *IFN-γ* genes in human PBMC and protected mice from the lethal effect of SEB [8]. Next, we showed that labeled SEB colocalizes fully with CD28 expressed on the surface of CD28-transfected cells, but does not bind to cells not expressing CD28, a strong indication that the superantigen binds directly to cell-surface CD28, even in the absence of MHC-II and TCR [8].

Direct binding of SEB to CD28 could then be demonstrated by surface plasmon resonance analysis [8]. This binding was specific, as the costimulatory ligand, PD-1, failed to bind SEB. Strikingly, CD28 bound not only to SEB but equally well to the 12-amino acid peptide mimetic of the β-strand/hinge/α-helix domain of SEB, but by contrast, not to scrambled or mutant forms of that peptide [8]. This result showed that SEB uses its β-strand/hinge/α-helix domain to bind directly to CD28.

### 2.4. The Superantigen Binding Site in CD28 is the Homodimer Interface

CD28 functions as a covalent homodimer [29] in its interaction with B7. The extracellular domain of CD28 is a β-barrel with two functions: binding another CD28 molecule to generate a homodimer and binding B7 coligand, through a distinct domain. We next addressed the question, where the binding site for superantigen toxins is located in CD28. This was a difficult challenge. We took advantage of our earlier finding that a peptide mimetic of the β-strand/hinge/α-helix domain in SEB inhibits cytokine gene expression in human PBMC induced by a particular monoclonal antibody against CD28 [8]. We used phage display this time to map the CD28 epitope of this monoclonal antibody, speculating that it might be the site where the SEB mimetic peptide binds, and by extension, holo-SEB. CD28 is highly homologous to the related costimulatory receptor, CTLA-4 [9]. Our epitope mapping led to a surprising result: the sequence in CD28 recognized by the antibody aligned with a completely distinct sequence in CTLA-4 that constitutes one part of the CTLA-4 homodimer interface. This CD28 peptide (HVKGKHLCP) indeed showed potent SEB antagonist activity in human PBMC [8]. If this was not coincidental, we argued, then a peptide having an 8-amino acid sequence (SPMLVAYD) located well over 100 amino acids upstream in CD28, corresponding to the location of another rim of the composite homodimer interface in CTLA-4, might also be an SEB antagonist. This proved to be indeed the case [8]. Both of these CD28 dimer interface mimetic peptides (but not scrambled forms) bound SEB and SEA, blocked induction of *IL2* and *IFN-γ* mRNA in human PBMC by SEB, SEA and even by TSST-1, and protected mice from lethal SEB challenge [8]. We observed protection also from SPEA [30]. Finally, we synthesized three peptides covering additional contact residues in the CD28 dimer interface and found that each was an active SEB antagonist. Our finding that the superantigen binding site in CD28 is in its crystallographic homodimer interface [8] is surprising. More commonly, ligand binding induces receptor homodimerization needed for signaling, as for human growth hormone receptor [31] and erythropoietin receptor [32].

### 2.5. The New Superantigen Synapse

**Figure 3:**
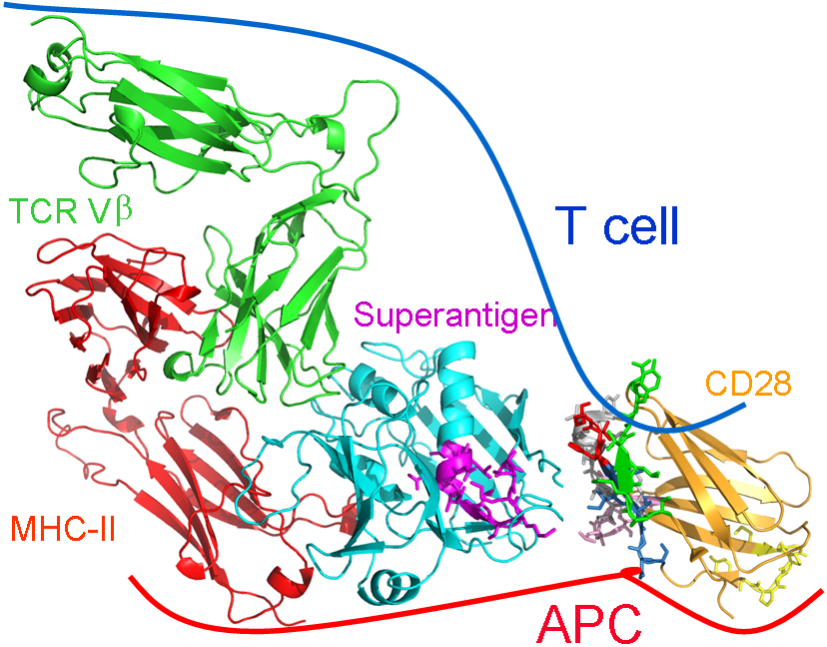
The superantigen synapse involves direct binding of toxin to CD28 at its homodimer interface. Structural model for Th1 cell activation by superantigens through simultaneous binding to three receptors: CD28, MHC-II and TCR. SEC3 (cyan), a close relative of SEB, in complex with TCR Vβ (green) and MHC-II (red) extracellular domains (1jck.pdb; [24]). The β-strand/hinge/α-helix domain in magenta (TNKKKVTAQELD in SEB) is freely accessible to the N-terminal 118-residue portion of the CD28 extracellular domain (1yjd.pdb; [29]). CD28 homodimer interface peptides are shown in color: red, HVKGKHLCP (only HVK is resolved in 1yjd.pdb); green, SPMLVAYD; pink, HKGLDSA; grey, YVNQTDIY; and blue, NGTII in NGTIIHVKG. The B7 binding domain MYPPPY is yellow. CD28 and TCR are oriented such that their trans-membrane domains (not shown) can enter the T cell at top; the MHC-II is oriented towards the APC at bottom. Modified from [8].

The results detailed above showed that binding of a superantigen to CD28 is critical for its function. Blocking access of the superantigen toxin to its CD28 receptor suffices to block its ability to induce the expression of inflammatory cytokine genes and lethality *in vivo* [8]. We now can redefine the superantigen synapse (Figure 3). Simultaneous binding of the superantigen molecule to CD28, TCR and MHC-II receptors is needed to produce the potent signal for Th1-type cytokine induction that leads to toxic shock [8]. Thus, superantigens make unconventional use not only of MHC-II and TCR through direct binding but also of CD28. The superantigen engages the CD28 dimer interface. The dimer interface has no known role in costimulation and is remote from the B7 binding site (Figure 3).

### 2.6. The Importance of Moderate Binding Affinity

Surface plasmon resonance binding analysis revealed that the superantigen engages CD28 with micromolar affinity [8]. Indeed, earlier studies had shown that binding of superantigen to the MHC-II and TCR receptors also occurs with micromolar affinity [33,34]. Thus, the superantigen toxin binds all three receptors with similarly moderate affinity. It is the concerted interaction with all three receptors, rather than an unusually high receptor affinity, that is responsible for the massive hyperactivation of inflammatory cytokine expression by the superantigen.

We were able to demonstrate this in the following manner. We mutated two amino acids within the β-strand/hinge/α-helix domain of SEB to alanine. The mutant SEB thus generated lost the ability to induce the inflammatory cytokines IL2, IFN-γ and TNF-α or to kill mice yet fully retained the ability to induce IL10, a cytokine that, we have shown [8], does not depend on signaling through CD28. This validated that the mutant toxin had retained full ability to signal through the TCR. What was the reason for the lack of biological activity of the mutant SEB? When analyzed by surface plasmon resonance, the mutant protein showed at least 5-fold higher affinity for CD28 than wild type SEB and it bound the CD28 dimer interface mimetic peptides likewise with far higher affinity. Moreover, the mutant SEB inhibited the action of wild type SEB, exhibiting a dominant-negative phenotype [8].

These results show that to be productive, binding of the superantigen to CD28 must be with moderate affinity and within the same range as its affinity for TCR and MHC-II. Only then can a synapse be created through concerted interaction, leading to powerful signaling into the T-cell. Indeed, mutant superantigens having higher affinity for the TCR can induce stronger T-cell responses yet superantigens invariably evolved as weak TCR binders [35], apparently to avoid loss of cooperative binding to MHC-II and CD28 through single occupancy of these receptors [8,35].

Peptide mimetics of the contact domains in superantigen and CD28, respectively the β-strand/hinge/α-helix domain in the superantigen and the homodimer interface in CD28, bind the opposite partner protein also with micromolar affinity [8]. The moderate affinity of the superantigen for each of its three receptors, including CD28, can explain why a short peptide, binding with similarly moderate affinity, can disrupt the immunological synapse and thus attenuate inflammatory cytokine induction.

### 2.7. The Remarkable Ability of Short Peptides to Compete with Intact Proteins

Potent superantigen toxin antagonism is displayed, *in vitro* and *in vivo*, by peptide mimetics of superantigen or of CD28. These peptides act as monkey wrenches that compete with the corresponding intact protein molecule in binding to its partner and thus prevent a productive interaction between superantigen and CD28 receptor. Indeed, peptides as short as 8 amino acids, as derived as from the CD28 dimer interface [8], can compete successfully in this setting. We were able to prove that this concept is correct: binding of SEB to CD28 expressed on the cell surface, in the absence of MHC-II or TCR, was abrogated in the presence of peptides mimicking either the β-strand/hinge/α-helix superantigen domain or the dimer interface of CD28; scrambling of peptide sequence abolished this ability, showing specificity for the active peptide [8].

The ability of such short peptides to mimic the full protein is surprising, particularly when considering the moderate affinity with which they bind to their target. We posit that the antagonist function relies on the ability of the short, largely unstructured peptide to undergo induced fit into the site occupied by the full protein it competes with, with the advantage that the peptide does not suffer from steric restrictions that modulate the fit of the intact protein molecule into its binding site. Although the full-size protein will occupy a larger contact surface in its target and be stabilized at multiple sites, obstruction of a single site by the competing peptide will suffice to destroy such cooperativity.

It should be emphasized that despite their potent superantigen antagonism, none of the peptide mimetics that we studied, whether of superantigen or CD28, had any detectable agonist activity by itself. This was manifested by a complete lack of induction of *IL2*, *IFN-γ* or *TNF-α* genes in human PBMC. Thus, whereas a short peptide is able to compete specifically with the full protein for its binding site, it does not mimic the biological activity of the full protein which relies on a far more complex set of interactions.

### 2.8. Potential Clinical Utility of a Superantigen Antagonist Peptide

To examine the potential clinical utility of our approach, we tested the ability of CD28 dimer interface mimetic peptide SPMLVAYD (colored green in the CD28 structure of Figure 3) to protect mice from bacterial infection with live superantigen-producing *Streptococcus pyogenes*. In this murine model of necrotizing soft-tissue infection, administration of a single dose of the peptide increased survival when given up to 5 h after infection, reduced inflammatory cytokine expression and bacterial burden at the site of infection, and improved muscle inflammation in a dose-dependent manner, without compromising cellular and humoral immunity [30].

## 3. Experimental Section

All peptides were synthesized with N- and C-terminal D-alanine residues in order to enhance their protease resistance. Experimental details were as described [1,8]. Cartoon models of protein structures in Figure 2 and Figure 3 were created in PyMol (www.pymol.org).

## 4. Conclusions

This work reveals a novel role for CD28, hitherto thought to function solely as costimulatory receptor in the immune response, as a direct receptor of a class of microbial virulence factors, the superantigen toxins.

We demonstrate that superantigen toxins must bind directly to CD28 in addition to the classical ligands, T-cell receptor and MHC class II molecule. The interaction of the superantigen with CD28 is critical for the ability of the toxin to elicit a massive inflammatory cytokine response having lethal sequelae. We identified the toxin binding site in CD28 as the receptor homodimer interface, and the CD28 binding site in superantigens as a compact domain that is structurally conserved among the diverse members of this broad toxin family produced by staphylococcal and streptococcal bacteria. This insight allowed us to design short peptide mimetics of these contact domains that by blocking access of the superantigen to CD28, effectively inhibit toxin action not only on human immune cells but also in intact animals. These peptides attenuate the inflammatory response sufficiently to allow for survival.

Our findings are relevant both in the medical arena where toxic shock is a major contributor to death and towards biodefense against potential terror/warfare use of these highly stable protein toxins.
